# Keeping all options open: Parents’ approaches to advance care planning

**DOI:** 10.1111/hex.12500

**Published:** 2016-09-26

**Authors:** Emma Beecham, Linda Oostendorp, Joanna Crocker, Paula Kelly, Andrew Dinsdale, June Hemsley, Jessica Russell, Louise Jones, Myra Bluebond‐Langner

**Affiliations:** ^1^ Louis Dundas Centre for Children's Palliative Care Institute of Child Health University College London London UK; ^2^ Marie Curie Palliative Care Research Department Division of Psychiatry University College London London UK; ^3^ Louis Dundas Centre for Children's Palliative Care Great Ormond Street Hospital for Children NHS Foundation Trust London UK; ^4^ Department of Sociology Anthropology and Criminal Justice Rutgers University Camden NJ USA

**Keywords:** advance care planning, children and young people, interviews, life‐limiting conditions, life‐threatening illnesses, parents

## Abstract

**Background:**

Early engagement in advance care planning (ACP) is seen as fundamental for ensuring the highest standard of care for children and young people with a life‐limiting condition (LLC). However, most families have little knowledge or experience of ACP.

**Objective:**

To investigate how parents of children and young people with LLCs approach and experience ACP.

**Methods:**

Open‐ended, semi‐structured interviews were conducted with parents of 18 children; nine children who were currently receiving palliative care services, and nine children who had received palliative care and died. Verbatim transcripts of audiotaped interviews were analysed following principles of grounded theory while acknowledging the use of deductive strategies, taking account of both the child's condition, and the timing and nature of decisions made.

**Results:**

Parents reported having discussions and making decisions about the place of care, place of death and the limitation of treatment. Most decisions were made relatively late in the illness and by parents who wished to keep their options open. Parents reported different levels of involvement in a range of decisions; many wished to be involved in decision making but did not always feel able to do so.

**Discussion:**

This study highlights that parents’ approaches to decision making vary by the type of decision required. Their views may change over time, and it is important to allow them to keep their options open. We recommend that clinicians have regular discussions over the course of the illness in an effort to understand parents’ approaches to particular decisions rather than to drive to closure prematurely.

## 1 Introduction

Over the course of an illness of a child with a life‐limiting condition (LLC), for whom cure is not likely, a variety of decisions must be made about care and treatment. Decisions may encompass the initiation, continuation or withdrawal of standard or experimental therapy including ventilation, changes in place of care and approaches to nutrition.^1^, ^2^ Various organisations have recommended that families and healthcare professionals (HCPs) have early discussions about goals of care, treatment options and plans for future care, often referred to as advance care planning (ACP).^2^, ^3^, ^4^, ^5^, ^6^

Most studies on ACP for children have taken place in the United States. Some have shown that parents who have participated in ACP may perceive these discussions as helpful in several ways.^7^ Parents report that the process can be valuable in ensuring the best care and quality of life; it can allow adequate time and information to make decisions, communication about desired care, and offer peace of mind.^8^ In some instances, ACP may help parents to acknowledge where they would like their child to die and achieve this preference, so that the death may occur more peacefully.^9^ However, other studies have shown that most families have little knowledge or experience of ACP discussions for their child.^10^, ^11^, ^12^, ^13^, ^14^, ^15^, ^16^, ^17^ For example, parents of sons with Duchenne muscular dystrophy reported that they were not familiar with the concept of ACP and they had not had any discussions of plans for future care within the family or with HCPs.^11^

In this article, we report the findings of a pilot study to explore parents’ experiences in the United Kingdom (UK) of ACP discussions for children for whom cure is not likely. We aimed to increase our understanding of how parents approach and experience planning for their child's future care. We included: (i) parents of children with life‐limiting and life‐threatening illnesses who were currently receiving specialist palliative care and (ii) parents of children who had recently received such care and had died.

## 2 Methods

### 2.1 Sample

Our sample was drawn from a large metropolitan specialist palliative care service in the UK. The size and scope of the caseload provides a population aged 0‐19 years with a diverse range of life‐limiting conditions, ethnic and socio‐economic backgrounds.

The invitation, recruitment and sampling methods have been described in detail elsewhere.^18^ Two groups were invited to participate: (i) parents whose child was currently receiving services from the palliative care team (Group A) and (ii) bereaved parents whose child had received care from the palliative care team and had died 6‐10 months previously (Group B). Parents were excluded if they were currently or had recently been participating in other psychosocial research, were unable to communicate in English or unable to give informed consent. Eligible parents were invited to take part by the specialist palliative care clinician under whose care they were registered.

Ethical approval was granted on 15 June 2011 by an NRES Committee (11/LO/0710) and on 4 November 2011 by an NHS Foundation Trust (09NS06). Informed consent was obtained from all participants.

### 2.2 Interviews

Interviews were conducted by two experienced researchers (EB, JC) at a time and place of the parent's choice. Interviews were audio‐recorded, and field notes were taken to provide context. All parents were invited for a second interview, 12 weeks later, to explore further any issues raised but not fully addressed in the first interview, informed by an iterative process following the reading of transcripts. We used an open‐ended semi‐structured topic guide, developed from the literature^19^ and designed to allow participants to provide a narrative of their own experiences of ACP. This included: (i) recounting of the child's illness, (ii) their involvement in decisions made, (iii) how they experienced the decision making, (iv) when decisions were made; and (v) how decisions were documented. A copy of the interview guides is available from the authors on request. At the end of each interview, every parent was given a leaflet containing details of referral services should they need further support; the day after the interview, the researcher contacted each person via the phone or email to see how they were.

### 2.3 Data analysis

All interviews were transcribed verbatim by an independent transcription service and checked for accuracy by the interviewers (EB, JC). The follow‐up interview was conducted after preliminary analysis of the first interview. Following each interview, the transcripts and field notes were read and reread independently by the two interviewers (EB, JC). They added non‐verbal behaviour recorded in their fieldnotes to the transcripts. Meetings were held post‐interviews to facilitate reflection and reflexivity in relation to the interview process. After first interviews, each interviewer prepared, and the other members of the research team (MBL, PK) reviewed, the timelines of the parents’ experiences of ACP including at what point in the trajectory particular decisions were made or discussed (eg diagnosis, end of life, crisis) and the child's condition (eg stable, unstable). They also prepared and the research team reviewed summaries of the key issues raised by parents. These findings then informed the content of second interviews.

**Figure 1:**
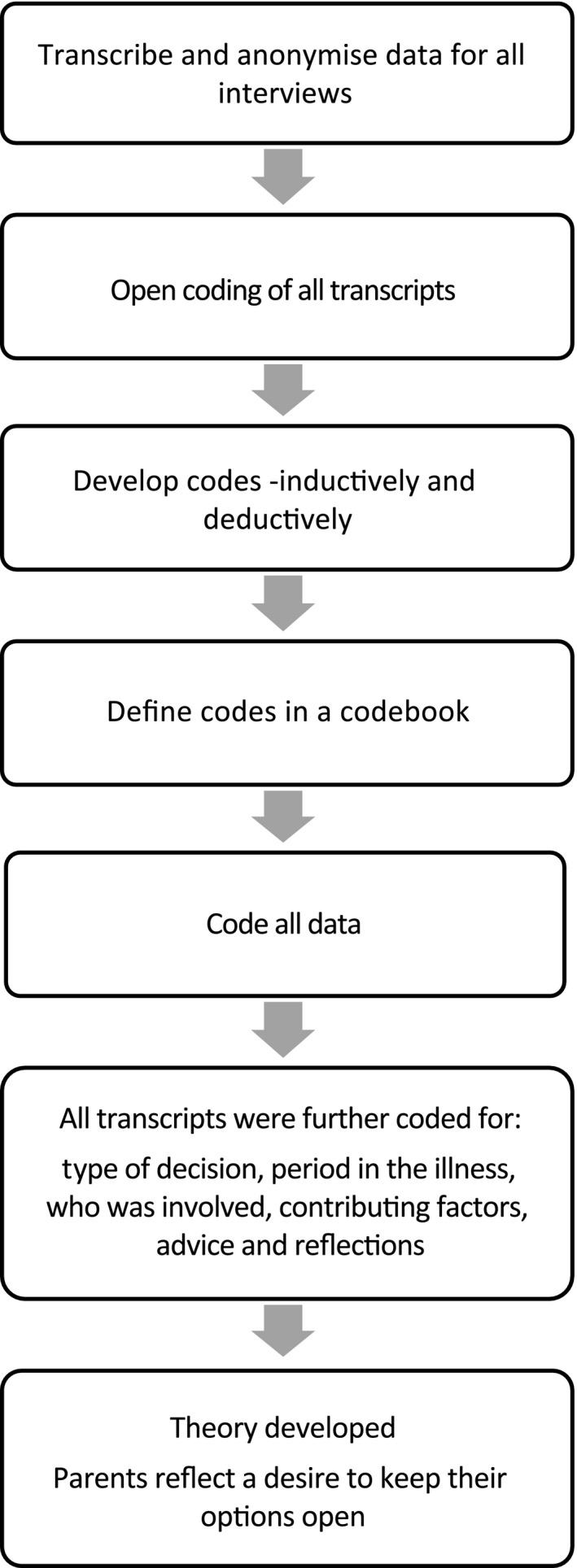
Overview of data analysis process

We sought to develop accounts of parents’ experiences of ACP and to uncover individual approaches. To do this, we used principles of grounded theory as described by Hennink, Hutter and Bailey^20^ which includes both inductive and deductive coding. This method was chosen to enable us to understand the experiences of this group of parents without the classificatory labels used by HCPs about ACP (inductive coding), while taking account of the context within which discussions and decisions might take place, for example the stage of the child's illness and those involved (deductive coding). At the close of data collection, using QSR NVivo 10 for qualitative analysis, members of the team (EB, JR) open coded the transcripts, line by line. The team (EB, JR, MBL) developed a codebook which included the open codes along with deductive codes from previous research.^21^ Definitions were developed for each code, and subsequently, the whole dataset was coded using the codebook. All transcripts were then consistently coded by members of the research team (EB, JR) for (i) type of decision made, (ii) periods in the illness and child's condition when decisions were made, (iii) who was involved in the decision, (iv) factors parents identified as contributing to the decisions made, (v) parents’ advice for other parents, (iv) reflections on participating in this or forthcoming studies. Our process is illustrated in Figure 1.

Apropos NVivo queries were conducted to explore relationships among the aforementioned codes/nodes in particular where the child was in the illness trajectory, the child's condition and who was involved (See Table 2).

## 3 Results

### 3.1 Participants

Between December 2011 and December 2012, parents of 519 living children (Group A) and 73 deceased children (Group B) met the inclusion criteria. Clinicians introduced the study to the parents of 28 (5%) children in Group A and 21 (29%) children in Group B and invited them to take part. These unexpectedly low invitation rates were explored in a separate study which revealed multiple barriers to invitation by clinicians. Barriers included infrequent contact with families and clinician concerns about families’ well‐being, and anticipated reaction to invitation^18^ and were confirmed in a study by Siden et al.^22^ Parents of nine children in each of Group A and Group B (total sample 18) consented to take part in an interview. Table 1 shows the characteristics of participants. Interviews were conducted between February 2012 and May 2013 and lasted a median of 80 minutes (range 19‐168 minutes). All parents were offered a follow‐up interview but for various reasons (eg death of the child or deterioration of their condition) not all parents could undertake a second interview. In six cases, parents completed one interview, in 11 cases parents completed two interviews and in one case parents completed three interviews at the family's request.

**Table 1:** Characteristics of parents who participated in interviews and their children

Characteristics	Group AParents whose child was currently receiving palliative care (9 cases)	Group BBereaved parents whose child had received palliative care (9 cases)
Children's characteristics		
Age group (yrs)^a^		
0‐1	1	1
1‐4	1	1
4‐12	3	3
12‐17	4	4
Sex		
Female	5	4
Male	4	5
Diagnostic group b		
Neurology	6	4
Gastroenterology	1	0
Metabolic	1	1
Chromosomal abnormality	1	0
Oncology	0	2
Immunology	0	1
Respiratory	0	1
Interview characteristics		
Interview participants		
Mother	7	6
Father	0	2
Mother and father	2^c^	1
Number of interviews with researcher		
1	4	2
2	4	7
3	1	0
Length of interview in minutes, median (range)		
Per meeting with the researcher	95 (19‐120)	63 (30‐168)
Total length	195 (19‐237)	105 (30‐315)
Interview location		
Home	5^d^	6
Tertiary hospital	2	2
Hospice	2^d^	0
Telephone	0	1

a For children in Group A, the age at the time of the interview is presented, whereas for children in Group B, the age at death is presented

b For purposes of confidentiality, the diseases affecting the children and young people are described according to the main International Classification of Diseases, 10th Edition (ICD‐10) 37 rather than by specific name of the disease or condition.

c One interview was conducted with a father and a stepmother.

d For one family, the first meeting with the researcher was in a hospice, while the second meeting took place at the family's home.

### 3.2 Findings from interviews

Parents’ accounts of their experiences of discussions regarding future care of their children, and making decisions about their child's care and treatment focussed on: (i) types of decisions made, (ii) periods in the illness and child's condition when decisions were made, (iii) who was involved in discussions and making decisions, (iv) their views of factors which contributed to those decisions, and (v) what they thought might be helpful for others.

- Types of decisions that were made

Parents reported having had discussions and making decisions about the place of care, place of death and limitation of treatment (see Table 2).

**Table 2:** Discussions and decisions reported by parents

Type of decision	Period in the illness	No of decisions	Details of discussions had and decisions made	Who was involved in each of the cases			
				Parents^a^	HCPs^a^	Others^a^	Not specified
Preferred place of care	Diagnosis	1	Hospice for respite	1	1	1 (SCP)	–
	Diagnosis/Unstable	1	Hospice for respite	1	1	–	–
	Stable	2	Taking the child home after birth/Hospice for respite	2	2	–	–
	Unstable	5	Hospice for respite/Taking the child home from hospital (2)/Finding the right place of care when the child could not go home (2)	5	5	–	–
	Unstable/End of life	1	Finding the right place of care when the child could not go home	1	1	–	–
	Crisis	5	Hospice for respite (2)/Transfer to (mental health) hospital/Care in residential school/Night care	5	5	2 (SCP, council, local MPs)	–
	End of life	2	Hospice for end‐of‐life care/Have the child at home near the end of life	2	2	–	–
	Not specified	8	Being at home as much as possible (6)/Hospice for respite or day care (2)	4	4	–	4
*Total decisions regarding preferred place of care*		*25*		*21*	*21*	*3*	*4*
Preferred place of death	Unstable	2	Transfer to hospice (2)	2	2	–	–
	Unstable/end of life	2	Bring the child home/Potential transfer from hospital to hospice	2	2	1 (SCP)	–
	Crisis	1	Transfer to hospice	1	1	–	–
	End of life	4	Transfer to hospice/Being home near the end (2)/Potential transfer from hospital to hospice	3	3	–	1
	Not specified	4	Staying home (4)	3	3	–	1
Total decisions regarding preferred place of death		13		11	11	1	2
Limitation of treatment	Resuscitation						
	Diagnosis	2	How to treat respiratory problems after birth/No aggressive interventions if the child deteriorated	2	2	–	–
	Diagnosis/revisited periodically	2	Not for resuscitation (2)	2	2	–	–
	Unstable	4	Up for full resuscitation/Not for resuscitation (3)	4	4	–	–
	Crisis	2	Not for resuscitation/Parents went back on previous decision not to resuscitate and asked to bag^b^ the child	2	2	–	–
	End of life	1	Whether to use aggressive interventions	1	1	–	–
	Not specified	3	Not for resuscitation (3)	2	2	1 (family friend)	1
Subtotal decisions regarding resuscitation		14		13	13	1	1
	Nutrition						
	Diagnosis	3	Fitting an NG tube (3)	0	3	–	–
	Stable	2	Performing a gastrostomy/Ordering milk	2	2	–	–
	Unstable	7	Fitting an NG tube/Performing a gastrostomy/Type of tube to be used for gastrostomy/Special diet/Pump feeds/How often to pass an NG tube when the child pulled it out/Having food for pleasure	7	7	–	–
	Unstable/Crisis	1	Hospital admission for TPN	1	1	–	–
	Crisis	1	Choosing between PEG and TPN	1	1	–	–
	End of life	1	Not to feed the child through TPN but let him deteriorate	1	1	–	–
	Not specified	2	Choosing between PEG and NG tube/Going back on TPN when NG feeds did not work out	2	2	1 (ill child)	–
Subtotal decisions regarding nutrition		17		14	17	1	–
	Other options for care and treatment						
	Diagnosis/unstable	1	Using a temporary rather than a permanent shunt	1	1	1 (extended family member)	–
	Unstable	9	Whether to treat a chest infection (2)/Whether to treat seizures/Making a care plan including a protocol for pain management/Making a care plan not to prolong the child's life unnecessarily/Limiting most interventions/Having all treatment available/Not to do a bone marrow transplant/Not to do a kidney transplant	9	9	1 (well sibling)	–
	Crisis	1	Whether to keep the child alive long enough for the family to say goodbye (due to family circumstances)	1	1	–	–
	End of life	3	Whether to use antibiotics/Whether to continue certain treatments in the last week of life/Whether to continue long‐term medication so the child would not wake up at the end of life	3	3	–	–
Subtotal decisions regarding other options for care and treatment		14		14	14	2	–
Total decisions regarding limitation of treatment		45		41	44	4	1
Total number of decisions		83		73	76	8	7

HCP, healthcare professional; NS, not specified; SCP, social care professional; NG tube, nasogastric tube (a tube that provides access to the stomach via the nasal passage); TPN, total parenteral nutrition (intravenous nutrition); PEG, percutaneous endoscopic gastrostomy (an endoscopic medical procedure in which a feeding tube is placed through the abdominal wall and into the stomach).

a Number of discussions/decisions in which these stakeholders were involved.

b Bag‐valve‐mask ventilation is a basic airway management technique that allows for oxygenation and ventilation of patients while avoiding more aggressive endotracheal intubation.

In 15 cases, parents recalled actively deciding where their child would be cared for, while in the other three cases parents either did not mention actively deciding or mentioned being happy with the place in which their child was cared for and/or contemplation of changes was not necessary. In 13 cases, parents reported discussions about using the hospice for respite and/or end‐of‐life care; however, most cases (n=13, eight Group A and five Group B) parents expressed a preference to care for their child at home as much as possible.

Discussions about preferred place of death were reported by parents of all deceased children in Group B and parents of four children in Group A (including parents of one child who had died before the follow‐up interview took place). Parents preferred their child to die either at home or in a hospice (n=12 cases, four Group A, eight Group B). The other five cases were Group A, and the place of death was not mentioned in the interview. Decisions were not necessarily straightforward. As one mother explained:

*“We wanted, well when she died she was at [the tertiary hospital], but we wanted to get her into the hospice, but we could…they, she wasn't stable enough to transfer her. That's where she wanted to be and where we wanted her to be, but we couldn't get her there.”* *—* *—*Mother of child 18 (Group B)

Discussions on limitation of treatment were mentioned by parents of all 18 children and included decisions about whether to undertake aggressive and invasive treatment, whether to treat infections with (intravenous) antibiotics, admit the child to the paediatric intensive care unit (PICU), assist breathing or intubate the child. A discussion of whether to attempt resuscitation was reported by parents of 14 children (eight cases in Group A and six cases in Group B). As noted by one of the mothers in Group B, discussions often involved what would happen during a resuscitation:

*“[the resuscitation officer] said, ‘What you see on TV is not what happens. No it really isn't. And I don't know how much sugar coating you want me to give you’, and like the dad was like, ‘I'm not into sugar coating. You know, I want you to tell me if my son stops breathing, what would you do to him?’ And they told him and they were graphic and in that room he said ‘No way. No’. He said ‘No, no, no’. He said, ‘Then we, we leave him’.”* *—* *—*Mother of child 5 (Group A)

Parents of 14 children (eight cases in Group A and six cases in Group B) mentioned discussions and decisions related to nutrition, including decisions about initiating, continuing or discontinuing total parental or enteral nutrition.

- Periods in the illness and child's condition when decisions were made

Parents mentioned different periods in the illness in which discussions were held and decisions made (Table 2). As shown in Table 2, decisions (n=10 decisions, 12% of all 83 decisions) were recalled as being made at the time of diagnosis and these were recalled in only eight cases. Most decisions (n=32, 39%), including those reported in 15 cases, were made at times when the child was unstable, and a change in intervention was required to address problems (eg inserting a feeding tube, or deciding about high dose chemotherapy). In addition, decisions (n=11, 13%) were reported to have been made in six cases when there was a crisis requiring urgent medical attention because of increasing symptom burden with worsening family distress, or as reported by parents in five cases at the end of life when death was thought to be imminent (n=14, 17%). Other decisions were not limited to a particular time period and seemed to be made repeatedly throughout the child's illness. For example, parents in two cases mentioned that written plans were revisited 6 monthly or annually.

Of note, many parents’ narratives indicated a desire to keep options open, by stating that they would decide at the time or by agreeing to limit treatment with the knowledge that they could change their mind later. Parents of only three children agreed to limit all active treatment; however, all with the proviso that they could change their minds, as illustrated in these remarks by one mother:

*“There's been many milestones. We sat in this very room some time ago where… it was deciding whether we continued to take [our son] into hospital for IV antibiotics. And it was decided that if I felt he was well enough and it was going to help, that we should do everything that we can… And we decided that we'd take every episode on its own merits.”* —Mother of child 2 (Group A)

Parents reported that it was difficult for parents to visualize the likely consequences of limiting treatment. Parents mentioned that making decisions about future treatment options was difficult because to their way of thinking care or treatment options were hypothetical and their preferences might change in the future as circumstances altered, as one father explained:

*“I sort of said, ‘Well, we're committing to a document that could be…’ and it was sort of eight months before she had died, ‘… where our feelings could change and… [the clinicians said]’ ‘Well, that's okay, we can rework it, you can re‐lodge it. And at any moment you can override the document verbally’. [..] I didn't really get the point of the document…Because you can override it and it's quite dynamic. And really, you write that document with your daughter still not showing the symptoms they will show when they're going to die.”* —Father of child 10 (Group B)

- Involvement in decision making

Parents reported a range of people involved in discussions and decisions about children's care and treatment, including the parents themselves, an ill child, a well sibling, an extended family member and a family friend, and health and social care professionals (see Table 2). However, most decisions (n=68, 82%) reported by parents did not involve anyone else apart from the parent(s) or the HCP(s).

Notably, parents reported that there were some decisions made within the family without consulting a HCP. Parents commented that sometimes HCPs asked parents to make a particular decision. Yet, as one mother explained parents did not always want the HCP to involve them in decision making:

*“The first major one, yeah. And decisions like when she has a chest infection, whether to treat or not to treat. And I had to decide that the first time in 2007. […] Em, so yeah, I found that quite harsh. If she was as ill as she is now then I'd understand, but at the time it was like, ‘Really? Well, no actually, yeah I want you to treat her’. So we did that.”* —Mother of child 3 (Group A)

By contrast, parents reported that there were decisions made by HCPs alone and sometimes treatment was implemented without a prior discussion with the parents, as one father commented:

*“And I think at that time he'd already had a nasogastric tube passed. [..] Em, so obviously that's how [..] some of his er, nutrition was being supplemented. [..] some of those decisions had been made without any input from us. [..] Well, had we… had we known, I think, em, that passing a tube was a significant step we may have asked for it not to be passed. [..] Because to then undo something isn't straightforward.”* —Father of child 11 (Group B)

Parents described different levels of involvement in decisions. Sometimes parents were happy to go along with the recommendation given by the HCP(s), or the HCP(s) went along with the parents’ preference, while at other times parents and HCPs jointly weighed the benefits and risks of the different options, as illustrated by one mother:

*“And he [the gastroenterologist] was the one really that we went and had a very lengthy discussion as to what all the pros and cons would be of doing this procedure, em, and whether he really thought it was the right thing to do.”* —Mother of child 17 (Group B)

- Factors identified by parents as contributing to decisions about the child's care and treatment

Parents reported that when making decisions about their child's care and treatment, they had considered several factors: some were focused on the ill child (eg in 12 cases parents mentioned their child not doing well/not responding to treatment as a factor in their decision making 28 times throughout all the interviews) or family as a whole (eg protecting other family members was mentioned 16 times in nine cases), others focused on reasons of their own or on advice given by HCPs. When deciding about the place of care and death, parents reported that they considered where the ill child would be most comfortable or have the lowest risk of infections, as well as the interests of any well siblings and their own capacity to care for the child and maintain family life. Wanting all interventions/treatments possibly available to the child could affect the place of care (eg a bone marrow transplant would require a long hospital stay).

When deciding about limitation of treatment, parents reported quality of life of the ill child as the major factor in their decision‐making process. Parents expressed conflicted feelings about these decisions because whereas they did not want their child to suffer, they also wanted to do everything possible to try to increase the length of their child's life. One mother described these feelings of conflict during her decision making:

*“Yeah I mean it was, it was awful. It's one thing talking about it, and I was like, ‘Yep, just bring it in, yep, let's get it done, yep, yep, yep’, and even reading it actually, ‘Yep, yep, yep’, [..] Erm, but actually going to sign it, it just, you know like, it just, ‘I'm so sorry [name of daughter]’, erm… even though it's the right thing, and even though it would have been awful having to turn a ventilator off, because she wouldn't have come off it, it's just that guilt thing you know, I'm signing to let her die which is just horrendous.”* *—* *—*Mother of child 14 (Group B)

Parents in eight cases reported being given strong advice by clinicians to limit treatment and why they accepted that advice despite misgivings. As one mother explained:

*“That's the difficulty is you're in a position where you know nothing and other people know a lot more than you. That doesn't always mean that they're right, but you have to kind of make a decision based on the information that you're given and therefore I don't know—was that the right decision or not? I don't know, but the fact is that [the palliative care nurse] thought it was. And therefore we accepted it as being the right thing to do.”* —Mother of child 16 (Group B)

Parents in eight cases felt they did not have much choice with regard to feeding options (eg because their child had an NG tube fitted directly after birth).

- Helpful ways to support parents when making decisions about the child's care and treatment

Parents discussed several ways that they thought would be helpful in supporting parents in decision making. All parents prominently mentioned the interaction between clinicians and parents, including the need for clinicians to understand the bigger picture of the life of the child and the life of the wider family rather than simply focusing on treating a particular symptom. Parents spoke of the importance of clinicians understanding the need for them to take professional control at certain times and provide practical help. Their suggestions also included the need for clinicians to give parents sufficient time to make decisions, allowing them time to adjust to the child's diagnosis or prognosis. In addition, parents mentioned it would be helpful to have more information about treatment options and likely outcomes, as one mother described:

*It wasn't actually probably until after he'd had his gastrostomy that I really understood what it means if you swallow into your airways and it actually goes down into your lungs. I just didn't realise before then. [..] it might have been actually a friend's, a friend who's got a little boy like mine, who actually explained it to me and said, ‘This is what…’ Yeah. And actually since then I've done that for a lot of parents. Because I didn't even know what it was going to look like*.—Mother of child 5 (Group A)

Finally, parents mentioned that it would be helpful if written plans were shared among all organisations involved with the care and treatment of the child, to reduce the paperwork burden and make sure the child receives care and treatment as agreed. One mother described this time‐consuming element of caring for her child:

*“It's lots of paperwork. It's lots of sitting down and doing the same paperwork over and over again. If you could do it once then that would be fantastic. [..] You have to do it for every agency that comes in, and for everywhere you go.”* —Mother of child 3 (Group A)

## 4 Discussion

### 4.1 Main findings

This study provides an overview of how parents of children with a LLC, cared for within specialist palliative care, approach and experience discussions of future care for their child, often referred to as advance care planning (ACP). We found that parents were involved in a variety of discussions and decisions about future options including the place of care and death, and limitation of treatment. Parents’ recollections of options discussed were very similar to those reported by parents in the United States^8^ and were in accord with the Institute of Medicine's recommendation that ACP discussions should include several aspects of end‐of‐life care, including place of care and death.^4^ Our findings confirm that for parents ACP is not just about withholding treatment or resuscitation status, but rather an on‐going conversation to create and revise a tailored, flexible plan considering daily life and end of life, to optimize the length and quality of life of the child.^8^, ^23^, ^24^

Most decisions were made relatively late in the child's illness. Similar findings emerged from a review of medical records of children with a LLC^25^, ^26^ and from interviews of caregivers of a child with cystic fibrosis, who recommended starting discussions about treatment options earlier.^17^ However, it is questionable whether the parents in our study would have welcomed earlier discussions, because even when ACP discussions were offered and held, most wished to keep their options open, and parents found it difficult to make decisions in advance of situations they still regarded as hypothetical. To accommodate families’ readiness to talk and their preferences, ACP plans should be flexible, allowing for possibilities to tailor the initiation, continuation and content of discussions.^17^, ^23^, ^27^

Parents in our study described how some plans and decisions were updated every six or 12 months, or when the condition of their child deteriorated. This is in line with a previous review of records of children with a LLC^24^ and with current recommendations for updating written plans.^28^ However, in addition to periodic updates, parents in our study reported a burden of having to repeat decisions in different environments. This burden could be alleviated if HCPs could document decisions in written plans and communicate these plans with others, including hospitals, schools, hospices and ambulance services. Recording and sharing the plan can help to provide practical guidance for HCPs and ensure that care and treatment follow the families’ expressed preferences.^23^, ^29^

As in other studies, parents reported a range of people having been involved in decision making at some point, including family members and friends, various HCPs and psychosocial professionals.^7^, ^23^, ^30^ While parents in this and previous studies indicated a preference to be involved in discussions and decisions,^10^, ^31^, ^32^ their chosen level of involvement is influenced by timing with regard to illness progression and the nature of the decision. Parents themselves have individual characteristics, and an approach that is both person‐centred and family‐centred is required. For example, as shown in this and previous studies, some parents perceived themselves to be the ultimate decision makers,^31^ while other parents preferred to participate or share in decision making but not to have the final say.^33^ A study in the Neonatal Intensive Care Unit has indicated an association between parents who perceived that they had shared in decisions and lower grief scores.^32^

We identified several ways to support parents in making decisions. Examples include, as also reported by parents in the United States and Australia, the sharing of information by clinicians in a trusting relationship,^34^ information about the potential outcomes of treatment options and the consequences of refraining from certain options.^10^, ^17^, ^35^ It might help parents to visualize the various treatment options or outcomes, if they are informed simply and shown pictures or videos or offered opportunities to see similar cases first hand.^36^ Our parents, and those in a previous study, appreciated clinicians providing a clear recommendation while still allowing parents to be involved in decisions at the level they prefer.^35^ In addition, we identified a need to provide parents with more information about the aims and process of ACP, including procedures for updating their preferences, and how written plans might be used.

### 4.2 Strengths and limitations

A primary strength of our work was the inclusion of perspectives from the parents of children with a range of LLCs, both deceased and alive, and not restricting the study to decisions about predefined options. The inclusion of a follow‐up interview for participants allowed researchers, guided by emerging data, to explore and understand the decision making process in more depth. Parents appreciated the chance to speak for a second time, as one mother put it: “it's almost like exploring it all, you know, like finding out different things about how it was. So it's been quite, quite good, like quite reflective.”

However, our sample was limited to the families of 18 children, and in most cases, only the child's mother participated in the interviews. An analysis of selection bias due to non‐invitation of eligible families has shown that clinicians were more likely to invite families they knew well, with whom they felt they had a “good” relationship.^18^ We appreciate that our sample was drawn from the caseload of a specialist paediatric palliative care team for whom ACP is a recognized aim of their practice; in other settings this may not be so.

That said, however, running throughout all of the interviews were expressions of both advantages and disadvantages of entering into discussions aimed at obtaining a decision about future care and treatment. Most importantly, parents expressed a desire to be allowed to keep their options open and not commit to plans of action that could not be revised at a later date.

Our findings suggest that to understand and take account of the nuanced nature of decision making for parents whose children face a limited future, further work should involve observation and recording of discussions between parents and clinicians as they occur in real time. We also note the need to capture the experiences of families for whom English is not their first language. This group is often excluded from research yet makes up a large proportion of the caseload of the palliative care team in a metropolitan hospital. We have now begun a longitudinal, prospective ethnographic study of children with high‐risk brain tumours to understand decision making in this context.

## 5 Conclusions

This study highlights that the views of parents of a child with a LLC change over time that their approach to decision making is influenced by the type of decision required and that most parents wish to keep their options open for as long as possible. As one mother told us: “Well, I don't know for how long she's going to live, but I have to live with the decisions I make”. We recommend that clinicians have regular discussions over the course of the illness in an effort to understand parents’ approaches rather than to arrive at a particular decision.

## Funding

This research received no specific grant from any funding agency in the public, commercial or non‐for‐profit sectors. The researchers were funded by the Louis Dundas Centre for Children's Palliative Care [grant number 2LGB/B (PK); 2LGB/C (LO); and 2LGB/E (JR)]; True Colours Trust [grant number 2LGA] (MBL); and Marie Curie Cancer Care [grant number MCCC‐FCO‐11‐U] (EB and LJ). The research was supported by the National Institute for Health Research Biomedical Research Centre at Great Ormond Street Hospital for Children NHS Foundation Trust and University College London.

## Conflict of Interests

No conflict of interests have been declared.
